# Assessment of Medical Students’ Knowledge of Ocular First Aid During Trauma: A Cross-Sectional Study From King Abdulaziz University

**DOI:** 10.7759/cureus.51843

**Published:** 2024-01-08

**Authors:** Lujain Idrees, Iman Wahby Salem, Ahella Jastanyah, Abeer Algarni, Razan Alsheikh, Abeer A Alyami, Leenah Alturkistani, Roaa Alnefaie, Sumiah Hijji

**Affiliations:** 1 Ophthalmology, King Abdulaziz University Faculty of Medicine, Jeddah, SAU; 2 Community Medicine, King Abdulaziz University Faculty of Medicine, Jeddah, SAU; 3 Faculty of Medicine, King Abdulaziz University Hospital, Jeddah, SAU; 4 Surgery, King Abdulaziz University Faculty of Medicine, Jeddah, SAU; 5 Medical School, King Abdulaziz University Faculty of Medicine, Jeddah, SAU; 6 Medicine, King Abdulaziz University Faculty of Medicine, Jeddah, SAU; 7 General Practice, King Abdulaziz University Faculty of Medicine, Jeddah, SAU

**Keywords:** saudi arabia, knowledge, medical students, trauma, ocular first aid

## Abstract

Background

Ocular trauma is defined as an eye injury of diverse types and subtypes, all of which can threaten vision. Hence, a precise first-aid approach is crucial to salvage the eyes and prevent blindness. Therefore, this study was designed to assess the level of knowledge among medical students at King Abdulaziz University (KAU) and identify factors that affect knowledge regarding ocular first aid in cases of trauma.

Methodology

A cross-sectional approach was used, targeting KAU medical students. First, students were asked to voluntarily respond to a previously used questionnaire that contained demographic data, including gender, marital status, socioeconomic status, academic year, and city of residence, followed by a history of ocular trauma. Then, questions were asked to assess their knowledge regarding different types of ocular trauma and proper first aid.

Results

A total of 310 participants responded to the questionnaire. Participants included both sexes, with 169 (50.8%) being males. Only 89 (26.7%) participants had a history of managing ocular injury. Knowledge levels were divided into good 76 (27%) and poor 201 (73%). Third-year students had the highest percentage of poor knowledge, whereas sixth-year students had the highest level of knowledge. The source of knowledge was mainly from studies.

Conclusions

The ocular first-aid knowledge level was predominantly poor, accounting for 201 (73%) of all responses. Thus, steps to enhance knowledge should be taken.

## Introduction

Ocular trauma is defined as injury to any eye structure. However, this definition relies on specific types of injuries, namely, mechanical eye trauma, chemical eye trauma, and infectious trauma. Ocular trauma is categorized into two main types according to the Birmingham Eye Trauma Terminology: open-globe and closed-globe injuries. Globe laceration and rupture are two subtypes of open-globe injury. The three types of lacerations include penetration, perforation, and intraocular foreign bodies. Similarly, lamellar laceration and contusion are two categories of closed-globe injuries [1].

Eye injuries must be managed with extreme caution because they can cause severe complications if left untreated. The goal of ocular first aid is to prevent scratching or further damage to the cornea. Therefore, ocular first-aid knowledge is crucial for responding rapidly to incidents. Hence, injuries can be recognized and managed effectively to preserve vision or provide the best possible outcome with minor degrees of damage before a skilled medical expert arrives to provide specialized treatment. Rapid recognition of eye injuries and early interventions are critical to ensure the best visual outcome. For example, in an observational study of over 11,000 eyes with serious injuries, 27% had <20/200 vision in the injured eye, described as legal blindness, and 61% showed visual improvement after receiving proper treatment [2].

According to the World Health Organization’s Blindness Data Bank, 55 million eye injuries occur worldwide yearly, of which 19 million result in vision loss or blindness [3]. Ocular trauma occurs at all ages; it has been shown that the majority are young males. Those between 22 and 44 years of age account for 10% of bilateral blindness cases; thus, these injuries may affect the vision of those patients for many of their remaining years [4].

A group of ophthalmologists presented a study to preview the magnitude of emergency department visits with complaints of eye-related injuries in the United States. The results showed a prevalence of 3.15 per 1,000 population [5].

The level of knowledge regarding ocular first aid can be assessed merely by asking the population about the correct action to be taken at the site of trauma in the most suitable form to obtain the best prognosis after therapy. For example, chemical trauma is best managed by immediately rinsing it with copious amounts of water before transportation to the nearest health facility [1].

An assessment of ocular first aid was performed among the general Saudi population in the region of Aseer in 2020, in which knowledge of the Saudi community regarding what to do at sites of eye injury was poor. The study emphasized that the worst level of knowledge was related to chemical injuries, which are the most dangerous types of trauma. This study highlighted the importance of a physician’s role in explaining the correct first-aid steps for traumatic eye emergencies [6]. Furthermore, another study conducted in Baghdad acquired ocular first-aid knowledge among emergency room nurses. It showed that regardless of the nurses’ sufficient information about eye injuries, they required additional education or training programs to enhance their expertise [7].

To determine how well the Saudi community knew about taking quick corrective action in eye injury cases, a cross-sectional study was conducted at King Abdulaziz University (KAU) Hospital in 2018. Data were gathered using a questionnaire and a random sample technique. These findings indicated that Saudi citizens need to be more knowledgeable about appropriate first aid in cases of ocular injury [7].

Given that no available studies have assessed the knowledge of all types of ocular injuries, this study aimed to evaluate the level of knowledge about first aid for different types of ocular trauma among KAU medical students. As medical students are future physicians, they must be aware of the tremendous effects of ocular trauma on patients’ vision. Therefore, patients should be aware of the appropriate actions to be taken immediately in cases of ocular trauma.

## Materials and methods

This cross-sectional study included all medical students (male and female) at the KAU Faculty of Medicine. This study was conducted at the Faculty of Medicine at KAU. It was approved by the Research Ethics Committee of KAU Hospital (reference number: 344-22).

A minimum sample size of 310 was calculated using the online Raosoft sample calculator [8], with a margin of error of 5%, 95% confidence level, and a population of 3,000 students. The pilot study was conducted with a sample size of 10% (31 students), which was excluded from the results.

A systematic random sampling was performed. Students’ lists for each year, both male and female, were obtained from the Deanship of Student Affairs, which included students’ phone numbers. Students were randomly selected from each level based on the K constant, and the questionnaire was distributed via the social platform WhatsApp to obtain the results. Messages provided the purpose of the study and links to the study, as well as gaining permission for participation. Once the student opened the survey link, a cover page containing detailed information about the study, the study’s title, the purpose of the study, and the time required to complete the questionnaire appeared. If they consented to participate in the study, they were asked to click “start the survey” and begin answering the survey questions. Participation was voluntary, and complete anonymity was ensured. The questionnaire has been previously used [6,9] and was constructed by a group of researchers based on the literature review, followed by a revision by three different experts to assure its validity. The questionnaire consisted of four components in English. The first component comprised demographic data, including sex, marital status, socioeconomic status, academic year, and city of residence. The second component included the history of ocular trauma, the third component included the questions assessing knowledge regarding the types of ocular trauma, and the fourth component included questions on proper first aid. The knowledge assessment included 17 questions; every question had only one correct answer, except for the question on cases where the participant had to go to the emergency room and symptoms of a scratched eye. The total knowledge score was 25. Participants were classified as having a poor knowledge level if they scored <60% of the correct answers for knowledge items and as having good knowledge if they scored ≥60.

Data were analyzed using SPSS version 26 (IBM Corp., Armonk, NY, USA). Qualitative data were expressed as numbers and percentages. The chi-squared test (χ^2^) was used to assess the relationship between variables. Quantitative data were expressed as mean and standard deviation (mean ± SD). Correlation analysis was performed using Spearman’s test. Statistical significance was set at p < 0.05. Finally, logistic regression was used to assess the relationships in this study.

## Results

The distribution of the students according to their demographics and injury history is presented in Table 1. Overall, 169 (50.8%) participants were male, and 325 (97.6%) were single. Of these, 74 (22.2%) were in their sixth academic year, and 312 (93.7%) lived in Jeddah. The majority, 217 (65.2%), had a monthly income of >10,000 SR, and only 89 (26.7%) had a history of injury.

**Table 1:** Distribution of participants according to their demographics and injury history.

Variable	N	(%)
Gender		
Female	164	(49.2)
Male	169	(50.8)
Social status		
Married	8	(2.4)
Single	325	(97.6)
Academic year		
Second year	6	(1.8)
Third year	66	(19.8)
Fourth year	69	(20.7)
Fifth year	64	(19.2)
Sixth year	74	(22.2)
Internship year	54	(16.2)
Residence		
Jeddah	312	(93.7)
Outside Jeddah	21	(6.3)
Monthly income (SR)		
<5,000 SR	40	(12)
5,000–10,000 SR	76	(22.8)
>10,000 SR	217	(65.2)
Injury history		
No	244	(73.3)
Yes	89	(26.7)

Table 2 shows the participants’ responses to the knowledge items related to ocular first aid during trauma. The results showed that 249 (74.8%) participants would go to the emergency room with persistent eye pain in cases of ocular trauma, 227 (68.2%) would go in cases of torn eyelids, and 272 (81.7%) would go in case of blood in the eye. Of these, 212 (63.7%) and 226 (67.9%) would visit the emergency room if they had an intraocular foreign body or a change in pupil shape, respectively. The most common correct symptoms of scratched eyes known by the students were eye pain (248, 74.5%), blurred vision (181, 54.4%), foreign body sensation (169, 50.8%), and light sensitivity (107, 32.1%). More than half of the participants, 190 (57.1%), correctly knew that the first thing to do in the case of a scratched eye was to rinse the eye with saline or clean water. Only 92 (27.6%) knew that they would blink more in the case of a scratched eye, and 231 (69.4%) disagreed with rubbing the eye to help remove any foreign objects. The majority, 287 (86.2%), correctly stated that in the case of scratched eyes, they would not wear contact lenses, and only 76 (22.8%) would not use redness-relieving eye drops. Approximately 142 (42.6%) knew that they would gently apply a cold compress in case of a blow to the eye. Of these, 230 (69.1%) knew that in the case of eye cuts or punctures, they would use a clean protective shield on the eye until seeing a physician, and 212 (63.7%) would not remove anything stuck in it. Most students, 278 (83.5%), knew that an injury could cause ocular complications, and 240 (72.1%) knew that washing with plenty of water was the first corrective action for chemical injuries. Approximately 201 (60.4%) knew that alkaline injuries were more dangerous than acidic ones, and 122 (36.6%) would not locate and remove particles in the case of chemical injury. More than half, 181 (54.4%), stated that they would not wash with an alkaline solution when injured with an acidic material, and 183 (55%) would not wash with an acid solution when injured with an alkaline material. The mean knowledge score was 12.62 ± 3.08.

**Table 2:** Distribution of the participants according to their responses to knowledge items related to ocular first aid during trauma.

Variable	N	(%)
In which case should you go to the emergency room?		
Will not go to the emergency	2	(0.6)
When you have persistent eye pain	249	(74.8)
When you have mild pain	53	(15.9)
When you get a vision problem	213	(64)
When you have torn eyelids	227	(68.2)
On eye movement not like the other	242	(72.7)
Blood in the eye	272	(81.7)
Presence of foreign body in the eye	212	(63.7)
Changes in the shape of the pupil	226	(67.9)
Which of the following are symptoms of a scratched eye?		
Eye pain	248	(74.5)
Feeling like something is stuck in your eye	169	(50.8)
Blurred vision	181	(54.4)
Light sensitivity	107	(32.1)
Darkness around the eye	33	(9.9)
Don’t know	31	(9.3)
If you get scratched in the eye, what is the first thing you will do?		
Clean the eye with cotton	8	(2.4)
I will close my eye	68	(20.4)
I will do nothing	25	(7.5)
I will rinse my eyes with saline or clean water	190	(57.1)
I will use anti-inflammatory eye drop	35	(10.5)
I will seek medical care	6	(1.8)
I don’t know	1	(0.3)
In case of a scratched eye, you have to blink more		
No	99	(29.7)
Yes	92	(27.6)
Don’t know	142	(42.6)
In case of a scratched eye, you have to rub your eye to help remove any foreign objects		
No	231	(69.4)
Yes	45	(13.5)
Don’t know	57	(17.1)
In case of a scratched eye, you can wear your contact lenses		
No	287	(86.2)
Yes	3	(0.9)
Don’t know	43	(12.9)
In case of a scratched eye, you can use redness-relieving eye drops		
No	76	(22.8)
Yes	76	(22.8)
Don’t know	181	(54.4)
What should you do in case of a blow to the eye?		
Gently apply a cold compress	142	(42.6)
Gently apply a warm compress	41	(12.3)
I don’t know	143	(42.9)
Put pressure on the eye	7	(2.1)
In case of cuts or punctures in the eye, apply a clean protective cover on your eye until you can see a doctor		
No	34	(10.2)
Yes	230	(69.1)
Don’t know	69	(20.7)
In case of cuts or punctures in the eye, you should remove anything stuck in the eye		
No	212	(63.7)
Yes	47	(14.1)
Don’t know	74	(22.2)
In case of cuts or punctures in the eye, which of the following is true?		
Avoid rinsing the eye with water	51	(15.3)
Nothing is true	79	(23.7)
Put pressure on the eye-protective covering	58	(17.4)
You have to take anti-inflammatory drugs	18	(5.4)
I don’t know	127	(38.1)
Chemical injury can cause ocular complications		
No	8	(2.4)
Yes	278	(83.5)
Don’t know	47	(14.1)
What should be the first corrective action when a chemical injury occurs?		
Cover the eye	5	(1.5)
For 15 minutes	1	(0.3)
Go to the emergency department	41	(12.3)
Pharmacy and eye drops	3	(0.9)
Wash with a little water	11	(3.3)
Wash with plenty of water	240	(72.1)
I don’t know	32	(9.6)
Alkaline injuries are more dangerous than acidic injuries		
No	48	(14.4)
Yes	201	(60.4)
Don’t know	84	(25.2
In case of chemical injury, you should locate and remove particles		
No	122	(36.6)
Yes	86	(25.8)
Don’t know	125	(37.5)
When injured with acidic material, wash with an alkaline solution		
No	181	(54.4)
Yes	32	(9.6)
Don’t know	120	(36)
When injured with an alkaline material, wash with an acidic solution		
No	183	(55)
Yes	29	(8.7)
Don’t know	121	(36.3)

Table 3 demonstrates the relationship between the participants’ knowledge level about ocular first aid during trauma and their demographics and injury history. A non-significant relationship was found between participants’ knowledge level about ocular first aid during trauma and their demographics or injury history (p > 0.05).

**Table 3:** Relationship between participants’ knowledge level about ocular first aid during trauma and their demographics and injury history.

Variable	Knowledge level				χ^2^	P-value
	Poor, N	Poor, %	Good, N	Good, %		
Gender					0.04	0.83
Female	95	47.3	37	48.7		
Male	106	52.7	39	51.3		
Social status					0.86	0.35
Married	4	2	3	3.9		
Single	197	98	73	96.1		
Academic year					9.30	0.09
Second year	6	3	0	0.0		
Third year	44	21.9	15	19.7		
Fourth year	44	21.9	10	13.2		
Fifth year	39	19.4	15	19.7		
Sixth year	36	17.9	24	31.6		
Internship year	32	15.9	12	15.8		
Residence					0.14	0.70
Jeddah	188	93.5	72	94.7		
Outside Jeddah	13	6.5	4	5.3		
Income					0.01	0.99
<5,000 SR	24	11.9	9	11.8		
5,000–10,000 SR	43	21.4	16	21.1		
>10,000 SR	134	66.7	51	67.1		
Injury history					0.05	0.82
No	148	73.6	57	75.0		
Yes	53	26.4	19	25.0		

**Figure 1:**
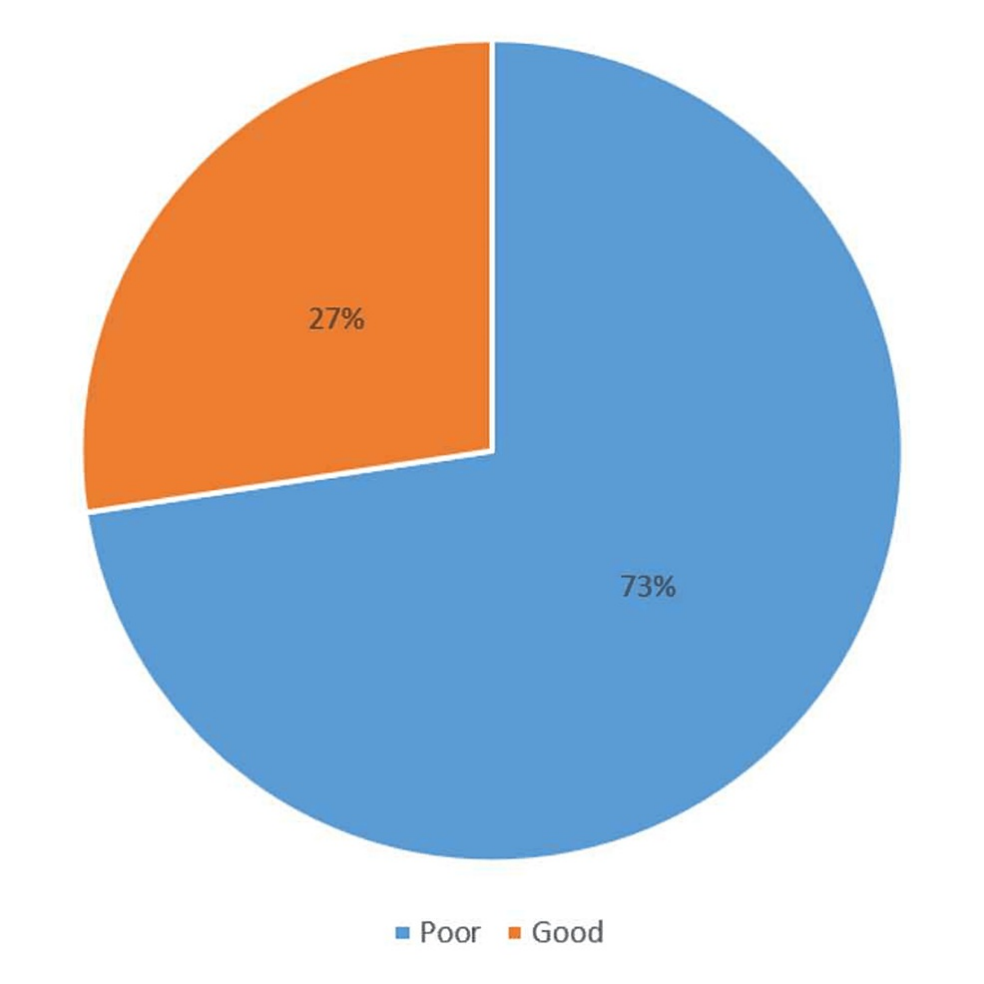
Percentage distribution of the participants according to their level of knowledge about ocular first aid during trauma.

Figure 1 illustrates the percentage distribution of participants according to their level of knowledge about ocular first aid during trauma. The prevalence of poor and good knowledge among participants was 76 (27%) and 201 (73%), respectively.

**Figure 2:**
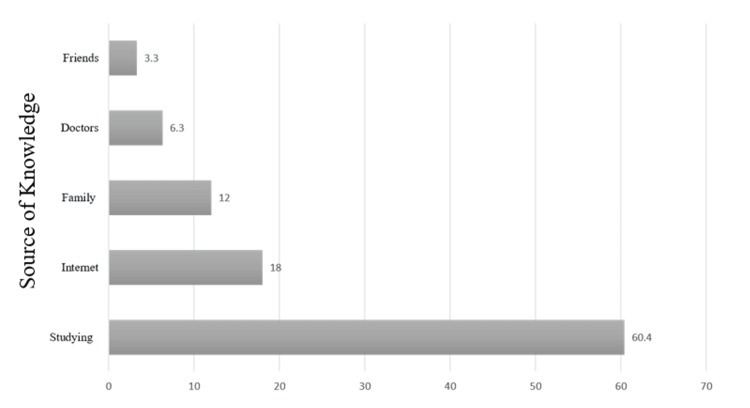
Percentage distribution of the participants according to their sources of knowledge about ocular first aid during trauma.

Figure 2 shows the percentage distribution of the participants according to their sources of knowledge regarding ocular first aid during trauma. The most common sources of knowledge about ocular first aid during trauma among participants were from studying (201, 60.4%) and the Internet (60, 18%).

**Figure 3:**
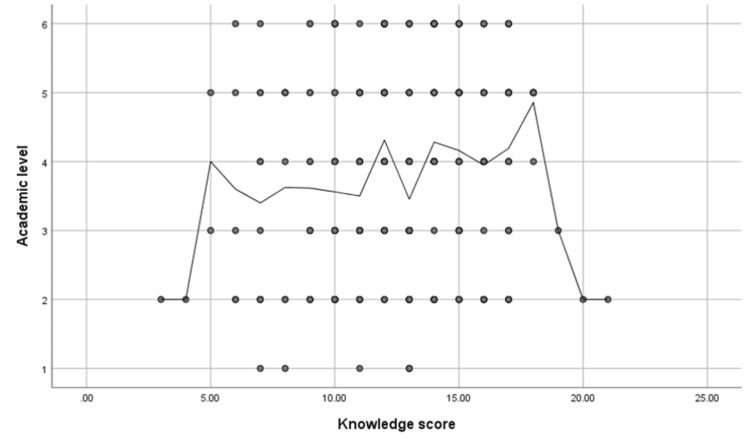
Spearman’s correlation analysis between knowledge score and academic level.

On the other hand, a significant positive correlation was found between knowledge score and academic level (r = 0.15, p = 0.01), as shown in Figure 3.

## Discussion

This study revealed that most medical students identified eye pain (248, 74.5%), blurred vision (181, 54.4%), foreign body sensation (169, 50.8%), and photophobia (107, 32.1%) as the correct symptoms of corneal abrasion. More than half of the participants (190, 57.1%) correctly identified irrigating the eye with saline or clean water as the first step in the event of corneal abrasion. A recent study in Saudi Arabia by Dhabaan et al. [6] reported that more than one-third (480, 39.6%) of their study population would rinse their eyes using saline or cold water in the event of ocular injury.

However, this study found that others would perform different measures, such as using ophthalmic anti-inflammatory drops and closing their eyes. Most students reported good awareness of the symptoms requiring emergency room visits. Regarding medical students’ awareness of action in cases of corneal abrasion, 287 (86.2%) avoided wearing contact lenses, and 231 (69.4%) would not have rubbed their eyes if they had symptoms of corneal abrasion. Regarding the use of decongestant drops in cases of corneal abrasion, this study found that 181 (54.4%) medical students did not know whether using these drops was the correct action. The remaining students agreed and disagreed equally with this step, indicating their awareness level concerning it. A study conducted by Souza et al. [10] in Brazil showed that decongestants will only procrastinate proper treatment for serious conditions. The American Academy of Ophthalmology has reported that over-the-counter decongestant drops can cause more pain. In addition, it does not aid the abrasion healing process. Thus, the use of any drop is not recommended before visiting an ophthalmologist [11].

Regarding blown eye injuries, this study showed that approximately 142 (42.9%) of the students did not know how to use cold compressors as a first-aid action. In the case of first aid for penetrating eye injuries, more than half of the students reported covering the eye with an eye shield and did not attempt to remove any foreign objects stuck in the eye until seeing an ophthalmologist, which is crucial to ensure that no further eye injury occurs. This finding was proven in another study by Mwangi and Mutie, which showed that proper coverage of the injured eye would prevent additional damage [12].

Regarding chemical injuries, more than half of the students were aware that the level of danger in alkaline damage is higher than that in acidic injury, and 240 (72.1%) medical students reported that the proper action that should be taken in this type of injury is vigorous irrigation with copious amounts of clean water. However, the misconceptions in this study were as follows: 29 (8.7%) students chose to wash with an acidic solution after being injured by an alkali, and 32 (9.6%) decided to wash with an alkaline solution after being injured by an acidic solution. In a previous study on chemical eye injuries among the general population in Jazan by Alqassim et al., [13] 40 (7.5%) participants believed that acidic solutions should be applied to alkaline burns, whereas 25 (4.7%) believed that alkaline solutions should be applied to acidic burns. There is a very high risk of exothermic reactions and, consequently, thermal damage in both washing methods. This study showed that, in cases of chemical injuries to the eyes, 240 (72.1%) medical students took the proper immediate action of vigorous washing with clean water. In contrast, 41 (12.3%) medical students believed that the first action should be to visit the nearest emergency room. The study by Alqassim et al. [13] revealed that 317 (59.1%) participants chose the correct immediate action, whereas 155 (28.9%) thought that the first action should be to go to the closest emergency room.

Chemical injury can lead to vision loss, which can significantly impact the quality of life. Regarding the knowledge of immediate corrective actions in cases of chemical eye injuries, 278 (83.5%) medical students agreed that chemical injuries can result in complications. Siraj et al. [9] concurred with this finding and found that 88% of people in the Saudi community were aware of chemical injuries and the appropriate course of action. Eyelid examination is essential after an ocular chemical injury to identify and remove solid particles that may be entrapped in the conjunctival fornices and serve as a reservoir for ongoing chemical release and inflammation [14]. However, this study found that, in cases of chemical injury, only 86 (25.8%) students agreed to eyelid examination, while 125 (37.5%) did not know whether a particle should be located and removed.

In contrast, Seraj et al. [9] found that 552 (62.2%) of their respondents preferred that particles should be identified and removed in the case of chemical eye injury. This study reported the effect of academic level on the knowledge score for ocular first aid in eye injury cases. There was a significant positive association between academic level and knowledge scores (p = 0.01). A descriptive study by Samir et al. [15] among 150 women with children who were secondary school graduates aimed to assess mothers’ knowledge and practices about eye trauma in early childhood. The level of education and knowledge of mothers were significantly correlated; most had inadequate information regarding eye injury, and more than half had insufficient levels of practice (73, 48.7%). This contrasts with the findings of Dhabaan et al. [6] who concluded that individuals with higher educational levels had more knowledge of proper ocular trauma first aid. This is also exhibited in this study, as sixth-year students had the highest percentages of good knowledge levels (24, 31.6%), as opposed to second-year and third-year students who displayed the highest poor knowledge level in equal percentages (44, 21.9%).

The current study found that having a personal history of ocular injury does not affect the awareness level of first aid regarding ocular injuries, as only 19 (25%) students with a good knowledge level had a history of ocular injury. This contrasts with the findings of Dhabaan et al., who reported that individuals exposed to multiple eye injuries were more knowledgeable about ocular trauma first aid [6]. This finding can be explained by the older demographic being exposed to various work environments and thus more subject to ocular trauma throughout their careers [13].

This study recognized several factors associated with a good level of knowledge, including gender, given that the acceptable level of knowledge was higher among the male population. The academic year in the Faculty of Medicine also demonstrated differences in the level of knowledge; the highest percentage of good knowledge was in sixth-year students, while fourth-year medical students comprised the majority of poor knowledge.

This study has certain limitations that should be taken into consideration. First, the study specifically focused on medical students from KAU, which may restrict the generalizability of the findings to other medical student populations. The results may not accurately represent the knowledge of ocular first aid among medical students from different institutions or countries.

Another limitation is the reliance on self-reported data. Participants’ responses may be subject to biases, such as overestimation of their knowledge or a tendency to provide socially desirable responses. This reliance on self-reported data introduces the possibility of response bias and may lead to inaccurate results.

While the study highlights the curriculum as the primary source of information for medical students, it is important to acknowledge that other factors could impact students’ knowledge levels. Individual study habits, personal interests, and exposure to clinical cases outside the curriculum may also influence their understanding of ocular first aid. These factors were not extensively explored in the study, limiting the comprehensive understanding of the determinants of knowledge levels.

Furthermore, the study’s cross-sectional design precludes the establishment of causality. Although the study identified a lack of knowledge among medical students, it cannot definitively establish a causal relationship between the curriculum and the observed knowledge levels.

By addressing these limitations, future studies can provide a more comprehensive understanding of the factors influencing knowledge levels and develop targeted interventions to enhance ocular first-aid knowledge among medical students.

In general, our study showed that only 76 (27%) of the study population had a good knowledge level, as good knowledge was defined as a score of 60% or more. Therefore, we recommend programs to raise awareness and promote proper management actions. Likewise, the reasons behind the different knowledge levels according to academic year are yet to be explored and warrant investigations in future studies.

## Conclusions

This study revealed that most KAU medical students’ knowledge regarding first aid for ocular trauma was unsatisfactory, comprising almost three-quarters of the study population at 201 (73%) students. The leading source of information for medical students was their curriculum, emphasizing the importance of providing better exposure to ocular first-aid management. Therefore, we recommend adding higher exposure to ocular trauma cases via simulation sessions and clinical scenarios and preparing students with the correct first aid steps that could preserve vision.
